# Safety First: A Comprehensive Review of Nutritional Supplements for Hair Loss in Breast Cancer Patients

**DOI:** 10.3390/nu17091451

**Published:** 2025-04-25

**Authors:** Andrea Sechi, Stephano Cedirian, Tullio Brunetti, Federico Quadrelli, Fernanda Torres, Antonella Tosti, Fabio Rinaldi, Daniela Pinto, Rolando Bolognino, Angelo Valerio Marzano, Bianca Maria Piraccini

**Affiliations:** 1 Dermatology Unit, Fondazione IRCCS Ca’ Granda Ospedale Maggiore Policlinico, 20120 Milan, Italy; 2 Dermatology Unit, IRCCS Azienda Ospedaliero, Universitaria di Bologna, Policlinico S. Orsola-Malpighi, 40138 Bologna, Italy; 3 Department of Medical and Surgical Sciences, Alma Mater Studiorum, University of Bologna, 40138 Bologna, Italy; 4 Private Practice, Rio de Janeiro 22410-000, Brazil; 5 Fredric Brandt Endowed Professor of Dermatology, Miller School of Medicine, University of Miami, Miami, FL 33146, USA; 6 Human Microbiome Advanced Project (HMAP), 20129 Milan, Italy; 7 Sapienza University of Rome, Unitelma Sapienza, 00185 Rome, Italy; info@rolandobolognino.it; 8 Department of Pathophysiology and Transplantation, Università degli Studi di Milano, 20122 Milan, Italy

**Keywords:** hair loss, telogen effluvium, chemotherapy-induced alopecia, breast cancer, targeted therapy, immunotherapy, safety profile

## Abstract

Among the distressing side effects of cancer treatments, hair loss is one of the most disturbing for the quality of life and adherence to therapy in breast cancer patients. Many patients take nutritional supplements to prevent hair loss or enhance regrowth. Based on their mechanism and timing of use, nutritional supplements could be divided into safe, cautious, debated, and contraindicated categories. Non-contraindicated supplements generally include safe supplements like vitamin D, which is not known to interfere with cancer treatments. Those that are contraindicated include phytoestrogens and compounds affecting estrogen pathways because of the risk of stimulating tumor growth in cancers sensitive to estrogen. Antioxidants like tocotrienols and resveratrol are given judiciously because of potential interference with cancer therapies dependent on reactive oxygen species. Supplements debated, including nicotinamide, folate, and iron, pose a risk by promoting cellular proliferation or altering the tumor microenvironment. Biotin is nontoxic but interferes with blood test results and is thus difficult in cancer monitoring. Evidence regarding nutritional supplements’ safety and efficacy in this context is conflicting. Management by an oncologist is required along with more studies to clearly establish the safety parameters and efficacy guidelines.

## 1. Introduction

Hair loss is considered the most serious side effect of chemotherapy among breast cancer patients and can manifest as chemotherapy-induced anagen effluvium (AE), endocrine-therapy-induced alopecia [1,2,3], telogen effluvium (TE), and permanent/persistent-chemotherapy-induced alopecia (pCIA) [1,2,3]. According to the Global Cancer Observatory, in 2022, the incidence of breast cancer was over 2.2 million cases, globally [1,2]. Of those who undergo chemotherapy, almost 8% of women refuse it for fear of hair loss [4].

The major safety concern with regard to ingredients in supplements targeting hair loss in women with active breast cancer treatment is the avoidance of estrogenic mimicking in cases of estrogen-sensitive cancers, or interaction with ongoing anti-cancer therapy [3,4,5]. This review examines the safety of various supplements used to treat hair loss, with a specific focus on their relevance to patients with breast cancer. The selection of supplements was based on their reported use and perceived efficacy in the management of alopecia, particularly chemotherapy-induced hair loss. A targeted literature search was conducted using the PubMed database, employing combinations of keywords such as “hair loss”, “alopecia”, “chemotherapy-induced alopecia”, “supplements”, “safety”, “adverse effects”, “cancer patients”, and “oncology”.

Although not a systematic review, we tried to include peer-reviewed articles that addressed both the effectiveness and safety profile of such supplements in the oncologic setting. Human subject studies, especially in breast cancer patients, and those that reported data on adverse effects, interactions, or contraindications in this patient population were given preference. Where clinical trials or large cohort studies were lacking, we emphasized the inconsistency and potential risks by citing both animal studies and in vitro findings.

## 2. Hair Loss in Breast Cancer Patients

Hair loss is rated as the most distressing side effect of chemotherapy by breast cancer patients, and it has been reported to be the reason that explains why almost 8% of women refuse chemotherapy altogether [6].

AE is characterized by acute hair loss during chemotherapy due to the action of the chemotherapeutic agents. It generally begins within a short time after the start of treatment, with the highest peak of hair loss occurring at about 6 weeks of chemotherapy, when anagen hair and hair density are at their lowest, followed by a plateau phase from weeks 6 to 18. After the cessation of chemotherapy, hair growth gradually returns to baseline within 3–6 months [6]. AE may be associated with telogen effluvium caused by stressful events, such as surgery or drugs [3,7,8]. pCIA was reported to occur in up to 40% of breast cancer patients treated with adjuvant chemotherapy, and it is defined as a total or incomplete hair regrowth 6 months following therapy completion.

EIA is defined as hair loss during the use of endocrine therapies, including selective estrogen receptor modulators (SERMs) or aromatase inhibitors [3,7,9]. In these cases, women typically complain of hair loss in a pattern distribution and changes in hair quality (texture and luster).

## 3. Supplements

Nutritional supplements are dietary products designed to provide additional calories, proteins, vitamins, and minerals to an individual, and are often available for oral administration in forms of tablets, capsules, gummies, liquids, powders or bars, among others. They may include one or more of the following ingredients:

- Vitamins (such as multivitamins or individual vitamins like vitamin D and biotin);
- Minerals (such as calcium, magnesium, and iron);
- Botanicals or herbs (such as saw palmetto);
- Botanical compounds (such as curcumin);
- Amino acids (such as tryptophan and glutamine);
- Biotherapeutics including live microorganisms (commonly referred to as “postbiotics”) and tyndallized probiotics;
- Oils (such as omega 3) [10].

Over the past decades, numerous over-the-counter hair supplements have been widely used as self-prescribed adjuvant therapies for hair loss, particularly among women [11,12,13,14]. The rationale of doing so is usually to increase levels of stimulators or decrease levels of inhibitors of hair growth. There is a large number of those supplements, with variable ingredients including multivitamins and other blends with herbs, herbal extracts, antioxidants, and amino acids. In fact, considering the daily recommended dose by the food regulatory agency in different countries, many products are in supraphysiological dosages of several ingredients, which is already a cause for concern even in a healthy patient [15,16].

The risks of supplements for hair loss in patients with breast cancer may be broadly divided into three categories, based on their mechanisms of action (Table 1):

- Estrogen Mimicking Effects: Some agents, such as phytoestrogens, act through binding with estrogen receptors alpha (Erα) or estrogen receptors beta (ERβ) and may stimulate the growth of ER-positive breast cancers [9,17,18,19]. Similarly, saw palmetto increases estrogen indirectly because 5α-reductase inhibition affects estrogen signaling. These supplements have a particular risk in patients with estrogen-sensitive malignancies.
- Increased Production of Estrogen: It is plausible that, in supplements such as pumpkin seed oil, beta-sitosterol may increase aromatase activity and thus convert androgens into estrogens, raising circulating estrogen levels. This results in the promotion of breast cancer cells through the overexpression of aromatase or estrogen receptors and makes such compounds contraindicated in estrogen-sensitive cancers [9,17,18,19].
- Antioxidant Effects: Antioxidants, such as resveratrol and tocotrienols, can neutralize reactive oxygen species (ROS) induced by chemotherapy or radiotherapy, which might reduce the efficacy of the treatments. Though antioxidants are generally good for health, their use during cancer treatments should be approached with caution, as they could counteract the therapy-induced oxidative stress [20].
- Cellular Proliferation and Tumor Microenvironment Effects: Supplementation with a variety of nutrients, such as folate, vitamin B12, nicotinamide (vitamin B3), iron, and zinc, can inadvertently support the development of tumors by providing substrate for DNA synthesis, cellular proliferation, or modification of the tumor microenvironment. For instance, iron fosters the growth of tumors by promoting the formation of ROS, and disturbances in the levels of zinc are known to take part in breast cancer [21].

Independent of the category, in an oncologic setting, it is always prudent to align every step with the oncologist for safe patient management [22].

To better assess the actual risks associated with nutritional supplement intake in women with breast cancer, we reviewed all available data in the literature. We categorized the supplements based on their nature and the specific phases of the therapeutic process in which they may be used: supplements that can be taken during chemotherapy, those indicated after chemotherapy, those with debated/insufficient evidence supporting supplementation, and those contraindicated in all stages (see Table 1 and Table 2).

## 4. Safe During Chemotherapy

Vitamin D is a fat-soluble vitamin synthesized by epidermal keratinocytes, and it is known to play an important role in modulating the growth and differentiation of keratinocytes by binding to the nuclear vitamin D receptor (VDR). It is deeply involved in various signaling pathways that control hair follicle growth and differentiation. In oncology patients, vitamin D deficiency is highly prevalent and linked to worse prognosis; plasma levels <20 ng/mL should be corrected, aiming for >30 ng/mL, often requiring higher-than-EFSA doses (up to 2000–4000 IU/day) under medical supervision [40]. A recent review, in which a vast amount of literature and several databases were analyzed, supported the association between vitamin D deficiency and not only an increased risk of developing breast cancer, but also a tendency toward more aggressive disease phenotypes [23,40]. However, there is no consensus on specific serum vitamin D levels that could lower breast cancer risk [40].

Vitamin B6 (pyridoxine) is a vital cofactor in over 100 enzymatic reactions, especially in amino acid metabolism, neurotransmitter synthesis (including serotonin, dopamine, and GABA), gluconeogenesis, and homocysteine metabolism [41,42]. In oncology, it is of particular relevance for three main reasons. First, its neuroprotective role: B6 is frequently used to prevent or attenuate chemotherapy-induced peripheral neuropathy, especially from platinum compounds (cisplatin, oxaliplatin), taxanes (paclitaxel, docetaxel), and proteasome inhibitors like bortezomib. While large-scale randomized controlled trials are still lacking, several observational studies and small cohorts have suggested that B6, alone or in combination with folic acid or alpha-lipoic acid, may reduce the severity and duration of neuropathic symptoms [41,42]. Second, B6 supports hematopoiesis, contributing to hemoglobin synthesis and bone marrow function. Deficiency, which can result from malnutrition, malabsorption syndromes, intensive chemotherapy, or chronic use of interfering drugs (e.g., isoniazid, methotrexate, certain antiepileptics), may lead to hypochromic normocytic anemia and disrupt homocysteine metabolism. Third, although generally safe at physiological doses, prolonged intake exceeding 100–200 mg/day may cause reversible sensory neuropathy (paresthesia, ataxia, impaired proprioception), potentially complicating the differential diagnosis in cancer patients already at risk of chemotherapy-induced neurotoxicity [41,42].

Recent preclinical and clinical evidence also suggests that B6 may help mitigate chemotherapy-induced alopecia [13,43]. D’Agostini et al. demonstrated that the combination of L-cystine and vitamin B6 prevented apoptosis-related alopecia in smoke-exposed mice, a model sharing pathophysiological similarities with chemotherapy-induced hair loss [44]. Additionally, a recent review highlights B6’s protective role against doxorubicin-induced alopecia, presumably by counteracting oxidative stress and follicular apoptosis [45]. Given its safety at appropriate doses and potential multi-faceted benefits, vitamin B6 supplementation could be considered a supportive strategy in selected oncology patients, including those experiencing hair loss, with careful attention to dosage and individual risk factors.

## 5. Safe Only After Chemotherapy

Tocotrienols are hydrophobic phenolic antioxidants, extracted from plant seeds, belonging to the vitamin E family, with a high affinity toward ERβ, especially γ and δ-tocotrienols, whereas they do not bind to ERα. These tocotrienols facilitate the translocation of ERβ into the nucleus to activate estrogen-responsive genes, such as Macrophage Inhibitory Cytokine-1 (MIC-1), Early Growth Response-1 (EGR-1), and Cathepsin D, in breast cancer cells expressing ERβ (e.g., MD Anderson-Metastatic Breast-231—MDA-MB-231) and those expressing both ER isoforms (e.g., Michigan Cancer Foundation-7—MCF-7). This binding induces changes in cell morphology, caspase-3 activation, DNA fragmentation, and thus apoptosis [21,28]. These observations are corroborated by a double-blind, placebo-controlled pilot study indicating that the addition of tocotrienols to tamoxifen might improve the latter’s performance regarding breast-cancer-specific survival due to its growth-inhibitory properties [17,18,19].

## 6. Debated Use After Chemotherapy

### 6.1. Debated Use After Chemotherapy Due to Cellular Proliferation and Tumor Microenvironment Effects

The safety of nicotinamide (niacinamide, vitamin B3) in cancer patients remains controversial. It lowers cholesterol levels in the cytoplasm and cellular membranes through nicotinamide-N-methyltransferase, increasing membrane fluidity in triple-negative breast cancer (TNBC) by inhibiting Protein Phosphatase 2A (PP2A) activity. This inhibition activates the downstream MEK/ERK/c-Jun/ABCA1 pathway, ultimately promoting an epithelial–mesenchymal transition (EMT) response [20]. It is well accepted that EMT allows the metastatic process in cancers of various kinds [20].

A clinical study analyzed the effect of niacin intake on 3504 cancer patients, showing a significant association of higher niacin intake with reduced mortality, particularly concerning cancer-specific survival [24]. For now, it should be not recommended in practical oncology until dose–response data are clarified.

Folate (folic acid, vitamin B9) is an enzyme cofactor participating in nucleic acid synthesis and in amino acid metabolism [16]. There are controversial studies about the influence of folate on the risk of breast cancer [46]. A case-control study on 129 cases of breast cancer and 271 controls estimated that any use of supplements containing folic acid at 400 mcg a day was associated with a reduced risk of breast cancer among women, especially in carriers of Breast Cancer gene 1 (BRCA1) mutations [46,47]. The opposite was reported by Kim et al. who showed that a high plasma folate (>24.4 ng/mL) concentration may be associated with an increased risk of breast cancer according to a prospective study in 164 BRCA1 and Breast Cancer gene 2 (BRCA2) mutation carriers [25,46].

Vitamin B12 is a water-soluble vitamin that plays the role of a cofactor for methionine synthase and affects the synthesis of DNA, RNA, and proteins; therefore, it is involved in the highly proliferative hair follicle cycle [16]. One observational study assessed the association of supplement use with breast cancer outcomes, using a cooperative group clinical trial among 1134 patients with breast cancer. Patients were queried about supplement use with a baseline-time-zero questionnaire and a follow-up questionnaire 6 months after [25]. It was found that the use of vitamin B12 before and during chemotherapy, which included various treatment schedules with doxorubicin, cyclophosphamide, and paclitaxel, was statistically associated with poorer disease-free survival and overall survival [26].

Vitamin B12 is a cofactor for methionine synthase, involved in DNA methylation; although high doses could theoretically promote cellular proliferation, there is no strong preclinical or clinical evidence of a direct pro-carcinogenic effect at commonly used supplemental doses. Its use should be limited to patients with confirmed deficiency (e.g., megaloblastic anemia, long-term metformin use), and empirical supplementation should be avoided in well-nourished oncology patients, with a prior assessment of B12, homocysteine, and MMA levels [26].

Further studies are required for understanding how the use of vitamin B12 both before and during chemotherapy can be associated with poorer outcomes [26].

Iron is a cofactor of ribonucleotide reductase, an enzyme involved in DNA production [48] and is an essential component for the synthesis of myoglobin or hemoglobin. The correlation between low iron stores and increased hair loss is poorly understood, and it is possibly related to the high sensitivity of rapidly dividing hair follicle matrix cells to even slight deficiencies in iron [48].

Iron, directly and indirectly, through tumor microenvironment effects, contributes to tumor initiation and progression: it allows for the generation of ROS and can interfere with antitumor immunity [26]. Moreover, tumors need huge quantities of iron in order to proliferate [26]. An observational study showed that supplementation with iron during and before various treatment schedules is highly associated with an increased risk of recurrence of breast cancer [26]. We suggest avoiding empirical use: patients with normocytic-normochromic anemia, chronic inflammation, or functional deficiency should be ruled out before starting supplementation.

The supplementation of zinc, an oligomineral involved in many enzyme pathways, in cancer patients is still controversial: indeed, some articles has shown how alterations in zinc homeostasis can contribute to the onset and development of breast cancer [21,27]. However, it remains unclear whether the risk is associated with zinc excess or deficiency. Moreover, zinc is frequently included in hair supplements at relatively high doses (greater than 15 mg/day), which may further complicate the assessment of its safety in this context.

Creatine is a nutritional supplement taken to enhance muscle mass and performance during high-intensity activity by increasing the availability of muscular phosphocreatine, which helps regenerate ATP. Creatine may promote the metastasis of breast and colorectal cancer through enhancing Glycine amidinotransferase (GATM) activity and upregulating Snail (SNAI2) and Slug (SNAI1), which fostered cell migration in orthotopic mouse models [28]. Although preclinical data suggest a potential pro-metastatic role in murine models, there are no clinical studies in humans confirming a direct oncogenic risk. To our knowledge, there are no studies regarding the involvement of creatine in hair loss, and creatine has no demonstrated effects on follicular growth or hair health. Given its association with metastasis in preclinical studies, its use is contraindicated. In the absence of human data and considering the high individual variability in metabolic responses to creatine, we recommend against its use in breast cancer patients, unless specifically indicated for nutritional support in sarcopenic contexts and under close clinical supervision.

Omega-3 and omega-6 have provided promising results for the treatment of hair loss [49]. A systematic review and an original research article investigated the protective effects of omega-3 fatty acids derived from fish consumption against breast cancer in Asian patients. The studies provided evidence supporting the potential of omega-3 fatty acids to reduce the risk of breast cancer in Asian populations [29,50]. However other studies showed that the evidence regarding the effects of omega-6, fish consumption, and omega-3 on all cancer outcomes is uncertain and has been assessed as being of very low quality [30,51]. The protective effect of omega-3 fatty acids (EPA/DHA) appears to be more solid in cancer prevention than in treatment. The dietary intake of omega-3 from natural sources (such as oily fish and fish oil) is considered safe and potentially beneficial. However, high-dose supplementation (>2 g/day) lacks strong evidence of efficacy in oncology and may interfere with coagulation, especially in patients treated with antiangiogenic agents. As for omega-6 fatty acids, there are currently no robust data suggesting oncological harm; however, the available evidence is limited by low methodological quality, and conclusions remain uncertain.

### 6.2. Debated Use After Chemotherapy Due to Limited or Insufficient Evidence

Pumpkin seed oil, thanks to its component beta-sitosterol, is able to inhibit 5-alpha reductase, blocking the conversion of testosterone to dihydrotestosterone (DHT) and therefore exerting an anti-androgen effect on both prostate and hair follicles, making it effective in treating benign prostatic hypertrophy (BPH) and hair loss in animal models [15,52]. Several studies have shown beta-sitosterol anticancer properties, including inducing apoptosis, inhibiting the proliferation of cancer cells, and modulating immune responses in various cancer types, such as breast cancer [31,53,54]. However, the possible increase of testosterone due to 5-alpha reductase inhibition may lead to an increase of estrogen levels through the aromatase pathway, putting these supplements among those banned in women with estrogen-sensitive breast cancer.

Whey protein, a high-quality supplement sourced from milk, is a complete compound that provides all nine essential amino acids necessary for muscle repair, recovery, and overall health. One investigation suggests that there may be a potential link between milk intake and the incidence of human breast cancer, which is more likely due to natural steroid hormones promoting mammary cell growth [32]. Although whey protein derived from cow’s milk is processed extensively to reduce its hormonal content, a potential risk for hormonal effects cannot be completely excluded.

Curcumin is a phytochemical derived from *Curcuma longa* (turmeric) that participates in arresting the cell cycle of breast cancer cells and in inducing apoptosis. Curcumin-loaded solid lipid nanoparticles arrest the cell cycle at G1/S and reduce the expression of cyclin D1 and Cyclin-Dependent Kinase 4 (CDK4), leading to the strong induction of apoptosis and ROS production in vitro; specifically, they induce apoptosis by activating P53 and P21 proteins, which regulate the Phosphoinositide 3-Kinase—Protein Kinase B (PI3K-AKT) and Nuclear Factor kappa-light-chain-enhancer of activated B cells (NF-κB) signaling pathways [33,55]. However, as no studies on humans are available and since curcumin may exert an anticoagulant effect by inhibiting the activities of Factor Xa (FXa) and thrombin, its supplementation may be hazardous in cancer patients [56].

The documented antitumor efficacy refers to nanoparticle- or piperine-enhanced formulations, not to dietary turmeric, which has low bioavailability. No randomized clinical trials in humans are currently available. Due to its anticoagulant effects, curcumin supplementation is not recommended during chemotherapy or surgical treatment.

Certain probiotic strains, such as *Lactobacillus acidophilus*, *Lactobacillus casei*, and *Bifidobacterium longum e Lactobacillus rhamnosus*, can effectively address common treatment-related side effects in adult cancer patients when used individually or in combination for at least four weeks. The benefits observed across these strains were similar, suggesting their potential to alleviate gastrointestinal issues, immune responses, and performance-related challenges [34,57]. Another recent meta-analysis from Qureshi and collaborators, analyzing data from fifteen studies involving 2197 participants undergoing cancer therapy, reported that while they seem beneficial in reducing the incidence of diarrhea, there is still a need for more comprehensive research to definitively assess their impact on other critical outcomes such as the rate of significant complications, surgical site infections, the length of hospital stay, and overall survival rates [58].

The inconclusive nature of these findings suggests that while probiotics might serve as useful supportive care agents, their role in broader clinical outcomes requires further investigation. Larger, well-designed clinical trials could help clarify their effectiveness and establish guidelines for their use in cancer care. As always, patients should consult with their healthcare providers before starting any new supplement, including probiotics, during their treatment.

The use of probiotics (e.g., *L. rhamnosus GG*, *B. longum*, *L. casei*) may improve gastrointestinal tolerance to oncologic treatments. However, there is no evidence that they influence disease progression or survival, and their supplementation should be evaluated on a case-by-case basis, especially in immunocompromised patients.

### 6.3. Regulatory Guidelines for Food Supplements in Cancer Therapy

The European Food Safety Authority (EFSA) plays a vital role in the formulation of guidelines and regulations concerning food supplements. These regulations cover key areas, including safety assessments, labeling standards, and the evaluation of novel foods and ingredients, all of which are particularly pertinent for those undergoing cancer therapies [59].

The EFSA’s assessments extend to the safety of particular dietary supplements concerning their health implications in cancer therapy. For example, the authority has evaluated cannabidiol (CBD) as a novel food but stresses the importance of additional data to fully ascertain its safety for cancer patients [60].

The EFSA has also highlighted the safety of certain dietary components, including omega-3 fatty acids, in the treatment and management of cancer [61].

## 7. Cautious Use After Chemotherapy

### Cautious Use Due to Monitoring Interference

Biotin (Vitamin B7) is a major cofactor for carboxylase enzymes and thus plays a role in gluconeogenesis, fatty acid synthesis, and amino acid catabolism. Biotin facilitates protein synthesis, especially keratin production, which is important for healthy hair and nail growth [62]. To our knowledge, no studies have explored the association between vitamin B7 supplementation and cancer risk. However, low-dose biotin intake (up to 10 mg) can interfere with immunoassays, including those used for tumor markers essential for monitoring and follow-up, such as Cancer Antigen 125 (CA 125), Cancer Antigen 15–3 (CA 15–3), and Carcinoembryonic Antigen (CEA) [35,63,64]. It is therefore advisable to instruct patients to stop biotin supplementation at least one week before blood tests.

## 8. Always Hazardous (During and After Chemotherapy)

### Hazardous Due to Hormonal Properties in Estrogen-Sensitive Cancers

Saw palmetto (*Serenoa repens*), derived from American dwarf tree berries, demonstrates a dose-dependent inhibition of proliferation ER-positive breast cancer cell lines because it induces apoptosis [36]. However, it has been shown that such effects are more specific to prostate-lines cells, making the safety debated and to be explored in breast cancer patients [37]. In addition, saw palmetto is a potent inhibitor of both types of 5α-reductase and increases the activity of 3α-hydroxysteroid-dehydrogenase, which promotes the conversion of 5α-DHT into its less potent metabolite, androstanediol [65]. 5-alpha reductase inhibitors may cause an increase in estrogen levels via the aromatization pathway and are, therefore, contraindicated in estrogen-sensitive cancers.

Phytoestrogens, due to their structural similarity with 17β-estradiol, may bind to estrogen receptors and thus show both estrogenic and anti-estrogenic impacts on breast cancer, especially on ER-positive subtypes. In ER-positive breast cancers, phytoestrogens may exert a promoting action on the proliferation of cancer cells through either ERα or G-protein-coupled estrogen receptor (GPER) signaling pathways [38]. Phytoestrogens have been documented to behave much like estrogen, and thus low concentrations may activate the growth of ER-positive tumors [66,67,68]. GPER, in particular, has been associated with the triggering of non-genomic pathways including EGFR/MAPK and PI3K/AKT, which may lead to a rise in the proliferation and fall of apoptosis in breast cancer cells [39]. On the contrary, some phytoestrogens, like soy isoflavones, have been demonstrated to have a protective influence, reducing the risk of the disease, most likely by affecting ERβ, which would exert anti-proliferative results in some subtypes of breast cancers [38]. Therefore, the effect of phytoestrogens on ER-positive breast cancer is complex and biphasic, and it strongly depends on factors such as dosage, type of receptor interaction, and patient-specific variables. One of the few phytoestrogens documented as safe in the literature is resveratrol [4,69]. Resveratrol is a naturally occurring stilbenoid with phytoestrogenic properties present in diets that exhibit high antioxidant, anti-inflammatory, and anticancer activities. It has been shown to interact effectively with GPER, thereby providing possible therapeutic effects in the case of breast cancer [39]. In murine models, it increases the expression of Nuclear Factor Erythroid 2-Related Factor 2 (NRF2), a major factor that protects against DNA damage in the breast [70]. Resveratrol does this by increasing the expression of NRF2-regulated antioxidant genes such as NADPH Quinone Dehydrogenase 1 (NQO1), Superoxide Dismutase 3 (SOD3), and 8-Oxoguanine DNA Glycosylase 1 (OGG1) while preventing E2 from suppressing detoxification genes such as Aldehyde Oxidase 1 (AOX1) and Flavin-Containing Monooxygenase 1 (FMO1) [70]. Furthermore, resveratrol triggers apoptosis and inhibits the progression of cancer in MCF-7 human breast cancer cells via a caspase-independent mechanism with the downregulation of B-cell lymphoma 2 (Bcl-2) and NF-κB [9].

Due to its antioxidant activity, which may potentially interfere with therapy-induced reactive oxygen species (ROS), resveratrol should not be taken during chemotherapy or radiotherapy. However, it may represent a promising post-treatment maintenance agent. Randomized controlled trials are currently ongoing.

## 9. Conclusions

Nutritional supplements have emerged as one of the most controversial areas in dealing with hair loss in patients with breast cancer due to various conflicting reports about their safety and efficacy. Though a number of them have been promising, other supplements have considerable risks, especially with estrogen-sensitive malignancies, in which compounds have the potential to act similar to or build up levels of estrogen, promoting tumor growth. This impossibility of definite recommendations for most supplements is a result of the paucity of well-designed and high-quality clinical trials. Additionally, our categorization of supplements was based on general safety trends but does not substitute for individual risk assessment. Genetic background, type of breast cancer, and stage of treatment must be considered in clinical decision making. Therefore, our classification serves as a conceptual guide, not an absolute recommendation. With these uncertainties, patient safety has to remain the top priority. All uses of supplements have to be weighed judiciously against possible compromise in the efficacy of treatment and the promotion of cancer progression in close cooperation with oncologists [4,5]. Future research will help in the further elucidation of the interaction of supplements, cancer biology, and oncologic therapies for safer and more effective care.

## Figures and Tables

**Table 1:** Studies on safety of supplement usage for hair loss in breast cancer patients.

Supplement	At the Diagnosis	During Chemotherapy	During Endocrine Therapy	During Post-Therapy Follow-Up
**Vitamin D**	S	S	S	S
**Tocotrienols**	S	H	S	S
**Niacinamide (B3)**	D	D	D	D
**Folate (B9)**	D	D	D	D
**Vitamin B12**	D	H	H	H
**Iron**	D	H	H	H
**Zinc**	D	D	D	D
**Creatine**	D	D	D	D
**Omega-3**	D	D	D	D
**Omega-6**	D	D	D	D
**Pumpkin Seed Oil**	D	D	D	D
**Whey Protein**	D	D	D	D
**Curcumin**	D	D	D	D
**Probiotics**	D	D	D	D
**Biotin (B7)**	C	C	C	C
**Saw Palmetto**	H	H	H	H
**Phytoestrogens**	H	H	H	H

**Legend: S** = Safe. **H** = Hazardous. **D** = Debated. **C** = Cautious use.

**Table 2:** Studies on supplements usage for hair loss in breast cancer patients.

Supplement	First Author/Year	Study Type	Outcome of the Study
Vitamin D	Ooi et al. (2010) [23]	Original article	An association between vitamin D deficiency and both an increased risk of developing breast cancer and a tendency toward more aggressive disease phenotypes.
Tocotrienols	Nesaretnam et al. (2010) [18]	Original article: double-blind, placebo-controlled pilot trial	Combining tocotrienols with tamoxifen may improve breast-cancer-specific survival due to their growth-inhibitory effects.
Nicotinamide (Vitamin B3)	Ying et al. (2022) [24]	Original article: retrospective cohort study	The study found a significant link between higher niacin intake and reduced mortality among cancer patients, particularly in terms of cancer-specific survival.
Folate (Vitamin B9)	Kim et al. (2016) [25]	Original article: prospective study	High plasma folate concentrations (>24.4 ng/mL) might be associated with an increased risk of breast cancer.
Vitamin B12	Ambrosone et al. (2020) [26]	Original article: observational study	The use of vitamin B12 both before and during chemotherapy is significantly correlated with worse disease-free survival and overall survival.
Iron	Ambrosone et al. (2020) [26]	Original article: observational study	Supplementation with iron during and before various treatment schedules highly associates, in fact, with an increased risk of recurrence of breast cancer.
Zinc	Sullivan et al. (2021) [27]	Original article	Alterations in zinc homeostasis can contribute to onset and development of breast cancer.
Creatine	Zhang et al. (2021) [28]	Original research article	Creatine may promote the metastasis of breast and colorectal cancer through enhancing GATM activity and upregulating Snail and Slug that foster cell migration in orthotopic mouse models.
Omega-3 and Omega-6 Fatty Acids	Park et al. (2012) [29]	Original research article	The study provided evidence supporting the potential of omega-3 fatty acids to reduce the risk of breast cancer in Asian populations.
	Engeset et al. (2006) [30]	Original article	The study found no association between fish consumption or marine omega-3 fatty acid intake and the risk of breast cancer.
Pumpkin Seed Oil	Vundru et al. (2013) [31]	Original article; In vitro study	This study has shown its anticancer properties, including inducing apoptosis, inhibiting the proliferation of cancer cells, and modulating immune responses in breast cancer.
Whey Protein	Fraser et al. (2020) [32]	Original article: cohort studies	The study suggests the incidence of human breast cancer, which is more likely due to natural steroid hormones promoting mammary cell growth.
Curcumin	Wang et al. (2018) [33]	Original article; in vitro study	Curcumin-loaded solid lipid nanoparticles arrest the cell cycle at G1/S and reduce the expression of cyclin D1 and CDK4, leading to the strong induction of apoptosis and ROS production in vitro; specifically, they induce apoptosis by activating P53 and P21 proteins, which regulate the PI3K-AKT and NF-kB signaling pathways.
Probiotics	Linn et al. (2019) [34]	Randomized controlled trial	Supplementation of probiotics is an easy and effective way to reduce the incidence and severity of radiation-induced diarrhea.
Biotin (Vitamin B7)	Kabiri et al. (2021) [35]	Original article	Biotin interferences have been observed in immunoassays used to measure cancer markers, which could pose a significant obstacle for monitoring and follow-up in these patients.
Saw Palmetto	Hostanska et al. (2007) [36]	Original article; in vitro study	Saw palmetto demonstrates a dose-dependent inhibition of proliferation ER-positive breast cancer cell lines because it induces apoptosis.
	Baron et al. (2009) [37]	Original article: in vitro study	This study has shown that such effects of saw palmetto are more specific to prostate-lines cells, making the safety debated and to be explored in breast cancer patients.
Phytoestrogens	Alnefaie et al. (2024) [38]	Case–control study	In ER-positive breast cancers, phytoestrogens may exert a promoting action on the proliferation of cancer cells through either ERα or GPER signaling pathways.
Resveratrol	Huang et al. (2023) [39]	Review	Resveratrol interacts effectively with GPER, thereby providing possible therapeutic effects in the case of breast cancer.

Abbreviations: Glycine amidinotransferase (GATM). Cyclin-Dependent Kinase 4 (CDK4). Phosphoinositide 3-Kinase—Protein Kinase B (PI3K-AKT). Nuclear Factor kappa-light-chain-enhancer of activated B cells (NF-κB). Estrogen Receptor Alpha (ERα). G-protein-coupled estrogen receptor (GPER).

## Data Availability

The authors confirm that the data supporting the findings of this study are available within the article.
